# Deep Learning Radiomics Features of Mediastinal Fat and Pulmonary Nodules on Lung CT Images Distinguish Benignancy and Malignancy

**DOI:** 10.3390/biomedicines12081865

**Published:** 2024-08-15

**Authors:** Hongzhuo Qi, Qifan Xuan, Pingping Liu, Yunfei An, Wenjuan Huang, Shidi Miao, Qiujun Wang, Zengyao Liu, Ruitao Wang

**Affiliations:** 1 School of Computer Science and Technology, Harbin University of Science and Technology, Harbin 150080, China; qihongzhuo@126.com (H.Q.); anyunfei@163.com (Y.A.); msdking@126.com (S.M.); 2 Department of Internal Medicine, Harbin Medical University Cancer Hospital, Harbin 150081, China; ppingliu@outlook.com (P.L.); dr_wenjuanhuang@outlook.com (W.H.); ruitaowang@126.com (R.W.); 3 Department of General Practice, The Second Affiliated Hospital of Harbin Medical University, Harbin 150001, China; wangqiujun2008@163.com; 4 Department of Interventional Medicine, The First Affiliated Hospital of Harbin Medical University, Harbin 150086, China; zengyao_liu@hrbmu.edu.cn

**Keywords:** pulmonary nodules, nomogram, mediastinal fat, benign, malignant, deep learning, multimodal fusion

## Abstract

This study investigated the relationship between mediastinal fat and pulmonary nodule status, aiming to develop a deep learning-based radiomics model for diagnosing benign and malignant pulmonary nodules. We proposed a combined model using CT images of both pulmonary nodules and the fat around the chest (mediastinal fat). Patients from three centers were divided into training, validation, internal testing, and external testing sets. Quantitative radiomics and deep learning features from CT images served as predictive factors. A logistic regression model was used to combine data from both pulmonary nodules and mediastinal adipose regions, and personalized nomograms were created to evaluate the predictive performance. The model incorporating mediastinal fat outperformed the nodule-only model, with C-indexes of 0.917 (training), 0.903 (internal testing), 0.942 (external testing set 1), and 0.880 (external testing set 2). The inclusion of mediastinal fat significantly improved predictive performance (NRI = 0.243, *p* < 0.05). A decision curve analysis indicated that incorporating mediastinal fat features provided greater patient benefits. Mediastinal fat offered complementary information for distinguishing benign from malignant nodules, enhancing the diagnostic capability of this deep learning-based radiomics model. This model demonstrated strong diagnostic ability for benign and malignant pulmonary nodules, providing a more accurate and beneficial approach for patient care.

## 1. Introduction

Lung cancer is one of the most common cancers in the world, with the highest mortality rate among malignant tumors, accounting for 23% of all malignant deaths [1]. The 5-year survival rate of patients with early lung cancer can be as high as 92% [2,3]. A previous study found that low-dose computed tomography (CT) screening detected more early-stage lung cancers compared to conventional chest radiographs, reducing mortality by 20% [4]. Thus, the early diagnosis of lung cancer is a key factor in improving cure rates and reducing mortality [5,6,7]. However, the early symptoms of lung cancer are insidious, and the most important imaging manifestations in the early stage are pulmonary nodules. Therefore, if pulmonary nodules, which are small growths in the lungs, can be detected and accurately diagnosed early, it can significantly improve the chances of recovery and greatly reduce the death rate from lung cancer [8].

Numerous studies have shown that adipose tissue inflammation is strongly associated with the development and progression of cancer [9]. Reactive oxygen species are produced because of inflammatory white adipose tissue accumulation, which then causes deoxyribonucleic acid damage to worsen [9,10,11]. In addition, the adipokines and extracellular vesicles released by adipose tissue accelerate tumor metastasis within the microenvironment [12,13]. Mediastinal fat is a type of visceral fat deposit in the chest cavity. It has been found that mediastinal fat can affect intrapulmonary tumor growth and invasion by altering the tumor microenvironment through the release of hormones, cytokines, and other signaling molecules [14].

Advancements in CT technology, reconstruction techniques, and low-dose chest CT screening have led to an increased detection rate of pulmonary nodules [15]. Previous studies have shown that radiomics can be used to analyze features in nodular images to build predictive models that reveal the relationship between features and tumor phenotypes [16,17]. Deep learning (DL) has made significant progress in automatically characterizing CT images. Recently, the successful application of deep learning techniques in medical image analysis has motivated many researchers to employ DL for pulmonary nodule classification. However, the accuracy of distinguishing between benign and malignant pulmonary nodules has not been entirely satisfactory [18,19,20]. In order to solve the above feature extraction problem, the concept of attention in human vision was proposed and applied to image classification and other machine learning tasks. Computer vision methods based on trainable attention mechanisms can effectively and autonomously focus on areas of interest for tasks, suppress irrelevant areas, and further improve the performance of DL models [21,22,23].

However, it is not well known whether mediastinal fat has a differential value for nodules. This study aimed to establish a prediction model based on the image features of pulmonary nodules and mediastinal fat and compare their diagnostic effectiveness. Additionally, we have chosen to utilize the nomogram as our prediction model due to its significant advantages. The nomogram can provide a visual tool that simplifies complex statistical models into a user-friendly, graphical format. This allows for the easy computation of probabilities and supports clinical decision-making by presenting individual risk assessments in a clear and interpretable manner. For instance, Balachandran et al. [24] have demonstrated the utility of nomogram in improving the predictive accuracy of clinical outcomes in oncology.

## 2. Materials and Methods

### 2.1. Participant Inclusion

**Figure 1:**
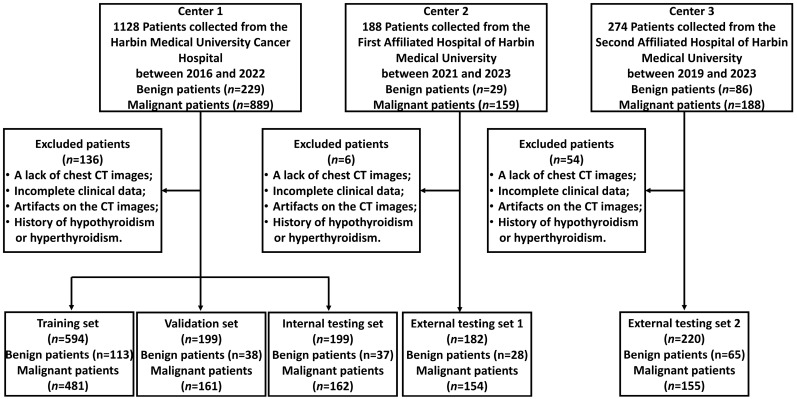
Flow diagram of the study population. Center 1: The Harbin Medical University Cancer Hospital; Center 2: The First Affiliated Hospital of Harbin Medical University; Center 3: The Second Affiliated Hospital of Harbin Medical University. Initially, we enrolled 1590 patients across three centers. Following the application of exclusion criteria, patients who did not meet the standards were excluded; specifically, 136 patients were excluded in Center 1, 6 patients were excluded in Center 2, and 54 patients were excluded in Center3. Ultimately, a total of 1394 patients were retained for the study. Among these, 992 patients from Center 1 were randomly divided into a training set, an internal validation set, and a test set. The remaining patients from the other two centers were allocated to external test set 1 and external test set 2, respectively.

This was a multicenter study, with a total of 1590 patients ultimately enrolled and treated. The clinical information of the patients was sourced from the hospital medical record management system, including sex, age, tumor history, pathological results, and other data. The study collected data from three centers: 992 patients from Center 1 (Harbin Medical University Cancer Hospital), 182 patients from Center 2 (The First Affiliated Hospital of Harbin Medical University), and 220 patients from Center 3 (The Second Affiliated Hospital of Harbin Medical University). All patients were divided into five sets: one training set, one validation set, one internal testing set, and two external testing sets. All patients underwent chest CT scans before surgery and received pathological results. The inclusion/exclusion criteria and patient recruitment process are shown in Supplementary File S1 and Figure 1.

The present study was carried out after approval from the Institutional Review Board of the above three centers. Because of the retrospective nature of the research, the requirement for informed consent was waived.

### 2.2. CT Examination

All patients from three tertiary first-class hospitals underwent chest CT examinations, with experienced radiologists from each center participating in data collection. The CT scan parameters are provided in Supplementary File S2 and Table S1.

### 2.3. Imaging Data Acquisition and Processing

**Figure 2:**
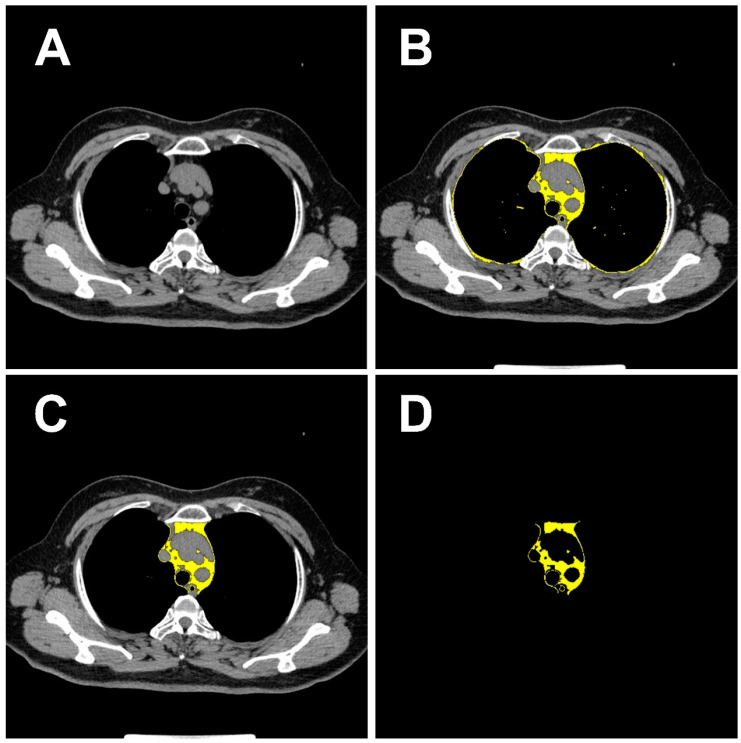
Process of extracting the mediastinal fat mask from computed tomography (CT) images. (**A**) Shows the original CT slice. (**B**) Represents the total intrathoracic fat tissue with Hounsfield Units (HU) threshold ranging from −200 to −40. (**C**) Represents the drawn region of interest for intrathoracic mediastinal fat. (**D**) Shows the mediastinal fat mask extracted from the CT image based on the mediastinal fat region. Initially, as shown in (**A**), the CT image is prepared at the level of the aortic arch. Then, as illustrated in (**B**), Image J is utilized to set a Hounsfield Unit (HU) threshold ranging from −200 to −40 in the CT image, which results in the delineation of the yellow region, encompassing all intrathoracic fat. Subsequently, as depicted in (**C**), the mediastinal fat region is manually delineated. Finally, the mediastinal fat mask is generated as shown in (**D**).

**Figure 3:**
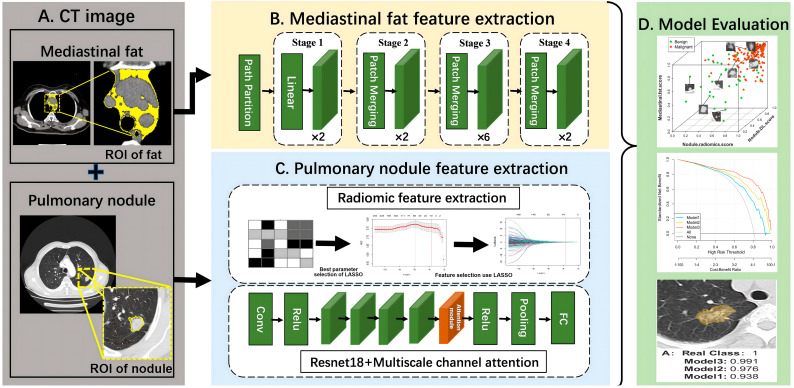
Model analysis process. (**A**) Data Preprocessing: Using specific areas in the CT images, namely the pulmonary nodule and mediastinal fat regions. (**B**) Mediastinal Fat Feature Extraction: Using the Swin Transformer model, a type of machine learning tool, to extract features from the mediastinal fat area. (**C**) Pulmonary Nodule Feature Extraction: Extracting radiomics features and deep learning features from the pulmonary nodule region separately. (**D**) Feature Fusion and Performance Evaluation: Fusing the mediastinal fat features and pulmonary nodule features to build the model and conduct performance evaluation. Initially, as illustrated in (**A**), the regions of interest (ROIs) for pulmonary nodules and mediastinal fat within the CT images are identified. Subsequently, as shown in (**B**), deep learning techniques are employed to extract features from the mediastinal fat. As demonstrated in (**C**), a combination of deep learning and radiomics are utilized to extract features from the pulmonary nodules. Finally, as depicted in (**D**), these extracted features are used to construct a predictive model and conduct performance validation.

For the pulmonary nodule region, the regions of interest (ROI-1), which are specific areas being studied in the CT images, were manually marked by an experienced radiologist on the central slice of the CT images with the largest tumor. For the mediastinal fat region, the Image J software version 1.53a (Wayne Rasband, National Institutes of Health, Bethesda, MD, USA) was used to analyze chest CT images at the level of the aortic arch and extract mediastinal fat tissue within the thoracic cavity. The software can segment tissue boundaries based on CT Hounsfield units (HU) values. Previous studies set the HU threshold for intrathoracic fat tissue between −200 and −40 [25,26]. An experienced radiologist selected the CT level at the first layer upward of the aortic arch and drew the region of interest to cover the mediastinal fat region (ROI-2) at that level, as shown in Figure 2. To ensure that the entire pulmonary nodule region and mediastinal fat region were captured, rectangular ROIs were cropped from the CT images based on the coordinates of the nodules and the location of the mediastinal fat before training the convolutional neural network (CNN). This operation allowed the regions of interest to adapt to the structure of the CNN. Additionally, all ROIs were normalized to achieve a standard normal distribution of image intensity. Detailed information on the processing of imaging data is shown in Supplementary File S3. To train the deep learning model and verify its robustness, Center 1 was randomly divided into a training set, a validation set, and an internal testing set in a 3:1:1 ratio. The training set was used to train the CNN model and the validation set was used to optimize the parameters of the deep learning model. The data from the other two centers were used as external testing sets. The workflow of the model is shown in Figure 3. Figure S1 shows the detailed process of extracting the mediastinal fat mask from CT images.

### 2.4. Radiomics Features Extraction of Pulmonary Nodules

Radiomics features of pulmonary nodules in CT images were extracted using the internal feature analysis program of Pyradiomics. For the radiomics model, the pulmonary nodule was used as the input. Radiomics features were extracted from the nodule mask and CT image, and then the most important features were selected for final classification [27]. The least absolute shrinkage and selection operator (LASSO) regression model was utilized to screen the radiomics features of nodules’ images. After LASSO feature selection, the selected nodule features were incorporated into the machine learning model for nodule malignant risk model construction. LASSO selected the most robust and non-redundant predictive features. Supplementary File S4 provides detailed information on radiomics feature extraction.

### 2.5. Deep Learning Feature Extraction of Pulmonary Nodules and Mediastinal Fat

In the pulmonary nodule network feature extraction, the ROI-1 of CT images were used as the input for a CNN model. We introduced a CNN with a multi-scale channel attention mechanism, using ResNet18 as the backbone, to extract features from pulmonary nodules. ResNet18 utilizes residual connections to add input features directly to output features across layers, enhancing the model’s ability to learn residuals. This architecture helps to preserve important details and mitigate feature loss in pulmonary nodule feature extraction. The presence of these connections allows for free information flow within the network, promoting feature reuse. By enabling direct communication between early and subsequent layers, ResNet18 efficiently passes low-level features to higher-level layers. This capability is particularly beneficial in extracting features like the shape, texture, and the edges of pulmonary nodules, thereby improving the consistency and stability of the features. In the network model, a multi-scale combined channel attention mechanism was used to learn the spatial relationships of the nodule regions in the CT images. Brief descriptions of the DL models are provided in the Supplementary File S5. The validation set data were used to fine-tune the model parameters to address the issue of overfitting in the DL model. The detailed CNN structure in this paper is shown in Figure S2. Subsequently, the output of the second-to-last fully connected (FC) layer of ResNet18 was used as the DL feature for each pulmonary nodule.

In the extraction of mediastinal fat network features, we employed the Swin Transformer model to overcome the limitations of traditional CNNs in handling large-size images. The Swin Transformer efficiently handles large-scale images through a hierarchical window-based self-attention mechanism. It processes pulmonary nodule images by dividing them into fixed-size blocks, which are sequentially processed through small Transformer blocks across multiple stages. This model emphasizes the local context within blocks and integrates broader contextual information by employing window-based self-attention, significantly reducing computational complexity. This includes a pooling stage that aggregates outputs from the Transformer blocks and refines them through downsampling operations, facilitating efficient global context modeling and the classification of pulmonary nodules. Each stage also features a Patch Merging layer that reduces spatial dimensions and enhances feature depth, optimizing the model for scalability and detailed feature extraction. By applying the Swin Transformer model to extract features from mediastinal fat images, we effectively captured global information in the mediastinal fat image, utilizing self-attention mechanisms and local window partitioning strategies. Detailed information about the Swin Transformer model is provided in Figure S3.

### 2.6. Score Building and Model Development

After feature selection, the deep learning radiomics scores for key features were compared using three different machine learning methods. As shown in Table S2, the logistic regression method performed the best. At the same time, the scores could be calculated for each patient. The association between the score and the malignancy of the pulmonary nodules was assessed in each dataset.

Multivariate logistic regression analysis was used to construct a nomogram from the features of the nodule region and mediastinal fat region. Backward step-wise selection was applied by using the likelihood ratio test with Akaike’s information criterion as the stopping rule. The combination of traditional radiomics features and deep learning features for pulmonary nodules can further improve the predictive performance of the model. Therefore, in this study, we first constructed a radiomics prediction model (Model 1) using radiomics features of pulmonary nodules. Next, we combined the radiomics features and deep learning features of pulmonary nodules to create a combined prediction model (Model 2) for comparison. Finally, we incorporated the extracted mediastinal fat mask features into the combined model to create an individualized nomogram (Model 3) for predicting the benign or malignant status of pulmonary nodules.

### 2.7. Models Performance Assessment

The overall performances of all models in this study were evaluated using the Brier score and Nagerkerke’s R^2^. The discriminative ability of the models was assessed using the C-index and the discriminant slope. Additionally, considering the imbalance of groups in the validation set, we performed 1000 rounds of Bootstrap sampling in each set for internal and external validation. Calibration curves and the Hosmer–Lemeshow test were used to evaluate the calibration of the models. The net reclassification improvement (NRI) and integrated discrimination improvement (IDI) were calculated to compare the performance of the models. To further quantify the clinical utility of the models, decision curve analysis (DCA) was performed by quantifying the net benefits at different threshold probabilities. The details of the model performance evaluation are provided in Supplementary File S6.

### 2.8. Statistical Analysis

In this study, we used R software (version 4.1.2) and SPSS software (version 26) for all statistical analyses to assess the relationships between different sets. The descriptive statistics were presented as means ± standard deviation (SD) for continuous variables and as percentages for categorical variables. Student’s *t*-test was employed to determine the difference between the two groups. The chi-square test was used for categorical variables between groups. A quantitative comparison of the C-index was carried out using the Delong test. All statistical tests were two-tailed, and *p* < 0.05 was considered indicative of a statistically significant difference.

## 3. Results

### 3.1. Clinical Characteristics

In this study, a total of 1394 patients with nodules were recorded, among whom 281 patients had benign pulmonary nodules and 1113 patients had malignant pulmonary nodules. The baseline characteristics of the patients were compared using *t*-tests and chi-square tests. Table 1 summarizes the characteristics of patients in the training set (*n* = 594), validation set (*n* = 199), internal testing set (*n* = 199), external testing set 1 (*n* = 182), and external testing set 2 (*n* = 220). Importantly, no statistically significant differences in baseline information were observed between the training and validation sets (Table 1).

### 3.2. Feature Selection and Score Building

A total of 289 radiomics features were extracted from ROI-1, along with 768 deep learning features extracted from ROI-2. The LASSO method, which is a statistical technique, was used to narrow down the number of features by reducing less important ones to zero, thereby selecting the key features from the training set and simplifying the data. Following dimensionality reduction, 10 radiomics features from the pulmonary nodule region and 9 features from the mediastinal fat region were selected, as illustrated in Figure S4.

We generated feature scores for ROI-1 and ROI-2 using logistic regression and standardized them within the range of 0 to 1. Specifically for the nodule region, we established separate radiomics feature scores (Nodule.radiomics.score) and deep learning feature scores (Nodule.DL.score). Additionally, to investigate the influence of mediastinal fat on the prediction of nodule benignity/malignancy, we further constructed an adipose score (Mediastinal.fat.score) using selected features from the mediastinal fat region. A detailed breakdown of the scores for these features are presented in Supplementary File S7.

### 3.3. Nomogram Construction

**Figure 4:**
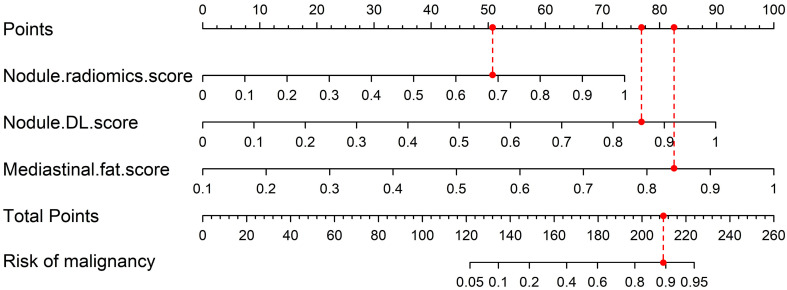
Model 3 construction and demonstration of use. Nodule region features and mediastinal fat region features are both incorporated into the nomogram. Model 3—a model that combines nodule region and mediastinal fat features. The dashed line in the figure is used to align the two parts of the scale.

The multivariate analysis of mediastinal fat and pulmonary nodule features is presented in Table 2, where Nodule.radiomics.score, Nodule.DL.score, and Mediastinal.fat.score were identified as significant predictive factors. Subsequently, we utilized logistic regression to integrate these factors. The algorithm automatically converted the regression coefficients into scale lines to construct the nomogram Model 3 (Figure 4) for predicting pulmonary nodule malignancy. Specifically, as depicted in Table 2, the coefficients of the three variables in the logistic regression model are 4.263, 5.182, and 6.411. These values are proportionally reflected in the lengths of the scale lines for each variable within the nomogram.

Additionally, we demonstrated how to use the nomogram with an example shown in Figure 4, involving a true-positive case of a 63-year-old male patient. Initially, the scores for the Nodule.radiomics.score, Nodule.DL.score, and Mediastinal.fat.score are 0.693, 0.857, and 0.845, respectively. These scores correspond to point values of 51.012, 76.133, and 82.581 on the Total Points scale line. After that, summing these points gives a total of 209.726, which is located on the Total Points scale line. Finally, aligning this value with the Risk of Malignancy scale line determines the patient’s probability of malignancy.

In order to assess the specific predictive value of mediastinal fat for pulmonary nodules, we also established and validated two comparative models that exclusively incorporated pulmonary nodule region features in the external testing set.

### 3.4. Validation of Nomogram Performance

**Figure 5:**
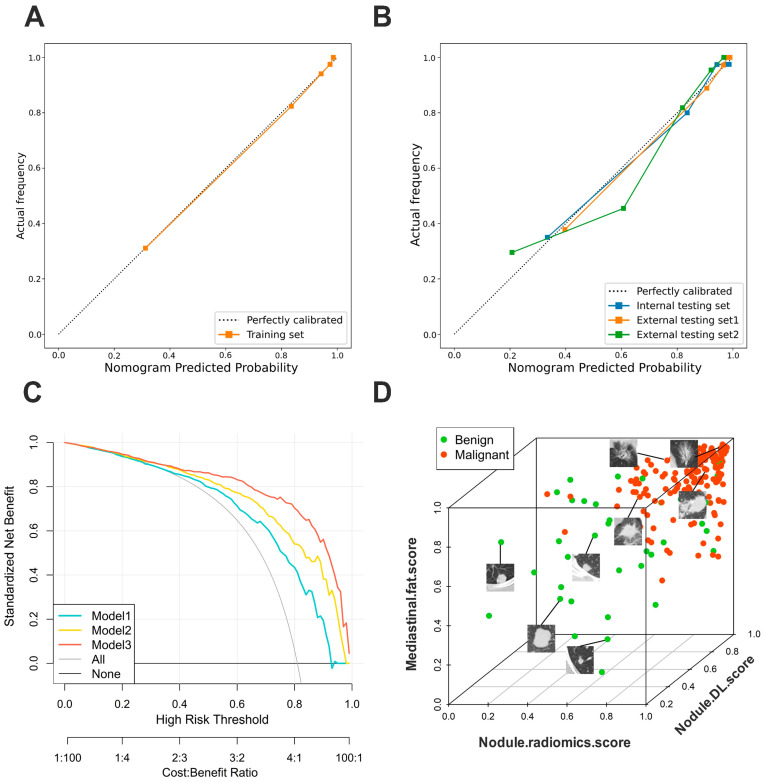
Clinical benefit evaluation of Model 3. (**A**) Calibration curves of Model 3 nomograms in the training set and (**B**) other sets; the calibration curve revealed a good predictive accuracy between the actual probability and predicted probability. (**C**) The predicted malignant probability is plotted on the X-axis and the observed malignant probability is plotted on the Y-axis. Decision curves for Model 1, Model 2, and Model 3. The X-axis and the bottom line display the risk threshold and the net benefit–cost ratio. The Y-axis shows the normalized net benefit for a wide range; the decision curves indicated that Model 3 could provide greater benefits to patients compared to Model 1, Model 2, “none” or “all” scheme. (**D**) The 3D visualization and scatter plot of the pulmonary nodule benign–malignant classification is displayed using scatterplot3d (version 0.3-41). Malignant nodules are highlighted in red and cluster in the upper right corner, while benign nodules are highlighted in green and are relatively scattered. Model 1—radiomics model that includes only nodule region features; Model 2—deep learning radiomics model that includes only nodule region features; Model 3—model that combines nodule region and mediastinal fat features.

Model 3 was the best for predicting the malignant pulmonary nodule, exhibiting good discrimination with C-indices of 0.903, 0.942, and 0.880 in the internal testing set, external testing set 1, and external testing set 2, respectively (Table 3). Furthermore, internal validation was performed across all sets. First, the calibration method with Bootstrap was used to demonstrate the correlation between actual and predicted probabilities, and the calibration curves were plotted, displaying good consistency between the predictions and observations for Model 3 in all sets (Figure 5A,B). The Hosmer–Lemeshow tests indicated no statistical significance (*p* > 0.05) in all sets, suggesting a well-calibrated model. In the bootstrapping validation, Model 3 also demonstrated high C-indices of 0.903 (95% CI, 0.843–0.962), 0.942 (95% CI, 0.901–0.983), and 0.880 (95% CI, 0.836–0.925) in the internal testing and two external testing sets, respectively. The C-index for deep learning features alone was 0.805, and when combined with fat features, it increased to 0.879. To assess the possibility of overfitting, the Delong test was performed on Model 3’s performance, revealing that the differences in C-indices between the training set and the three testing sets were not statistically significant, with *p* values of 0.557, 0.227, and 0.134, respectively.

### 3.5. Clinical Use

To assess clinical utility, decision curves were used to compare the benefits of Model 1, Model 2, and Model 3. It was observed that patients would derive greater benefits from Model 3 when the clinical decision threshold probability fell within the relevant range (Figure 5C). Furthermore, benign and malignant pulmonary nodules were visualized in 3D space, as demonstrated in Figure 5D. To further assess the predictive value of Model 3 in clinical applications, clinical impact plots and receiver operating characteristic (ROC) component plots are provided as Supplementary Materials, shown in Figure S5. As shown in Supplementary File S8, we extensively explored the sex, age, CT version, and CT image thickness groups of different patients and conducted Delong tests to assess the differences in model performance among the different subgroups. Additionally, we present detailed stratified ROC curves in Figure S6.

### 3.6. Incremental Predictive Value of Mediastinal Fat Region

**Figure 6:**
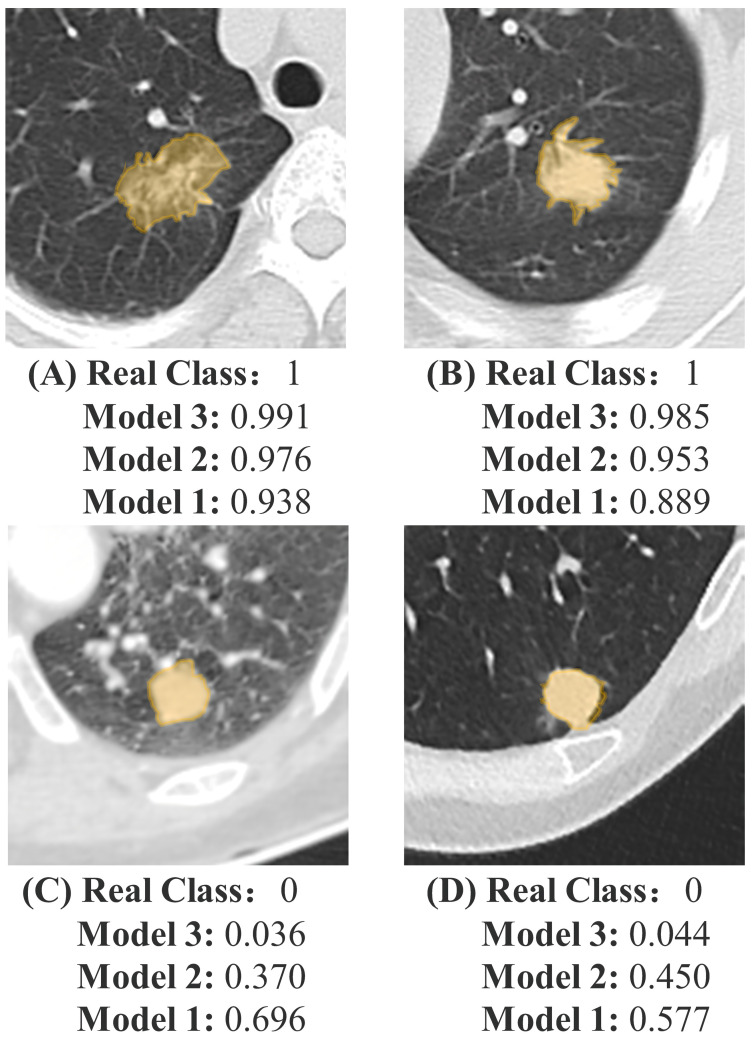
Examples of predictions using Model 1, Model 2, and Model 3. (**A**,**B**) represent a benign sample and (**C**,**D**) represent a malignant sample. The yellow region represents the nodule area. The models provide the predicted probability of malignant nodules. The real class, 0, represents benign nodules, and 1 represents malignant nodules. Model 1—radiomics model that includes only nodule region features; Model 2—deep learning radiomics model that includes only nodule region features; Model 3—model that combines nodule region and mediastinal fat features.

To further evaluate the impact of mediastinal fat tissue on predicting nodule performance, NRI and IDI were calculated, showing that Model 3 outperformed Model 2. According to the quantitative results, in the training set (NRI = 0.144, IDI = 0.130, *p* < 0.05) and in the internal testing set (NRI = 0.243, IDI = 0.090, *p* < 0.05), Model 3, which incorporated mediastinal fat region features, exhibited better predictive performance than Model 2, which relied solely on pulmonary nodule region features. The detailed results are shown in Table 4. Figure 6 displays some nodule images and their corresponding malignant prediction probabilities.

## 4. Discussion

In this study, we utilized datasets from three centers to establish models for identifying the malignancy of pulmonary nodules. The results indicated that the combined model, which incorporates both mediastinal fat and nodule regions, exhibited higher diagnostic performance compared to using the nodule region alone. To the best of our knowledge, this is the first analysis of predictive biomarkers incorporating mediastinal fat tissue for distinguishing between benign and malignant pulmonary nodules.

However, the mechanism of mediastinal fat involved in the prediction of malignant pulmonary nodules is unknown. White fat is an important endocrine and metabolic organ, as well as a key player in immunity and inflammation [28]. Fat depots in healthy lungs have a critical role in regulating alveolar lipid homeostasis and lung surfactant production [29,30]. Recent studies have observed that ectopic fat deposition could obstruct airways and aggravate lung injury [31,32]. Long-term, mild inflammation associated with obesity can lead to the development of scar tissue in fat and eventually promote cancer growth [33,34]. Increased proinflammatory adipose tissue macrophages are found in obese individuals [35,36]. The epithelial–mesenchymal transition and tumor immune escape may be triggered by these macrophages [37]. According to a report, colorectal cancer patients’ peritumoral adipose tissue underwent different morphological and functional changes as a result of a particular macrophage invasion. Another study established adipose tissue’s novel role in mediating the anti-cancer effects of cold exposure through brown adipose tissue activation [38]. A report identified distinct morphological and functional changes in the peritumoral adipose tissue caused by specific macrophage infiltration in patients with colorectal cancer [39].

In this study, incorporating mediastinal fat into the prediction model yielded a C-index of 0.903 (95% CI: 0.843–0.962), providing compelling evidence for the association between mediastinal fat and the benign/malignant classification of pulmonary nodules. At present, research investigating the relationship between pulmonary nodule malignancy and fat tissue remains limited. Some studies showed that mechanisms related to obesity could result in functional limitations within the respiratory system and patients with interstitial pneumonia exhibited thicker mediastinal fat compared to individuals without the condition [40,41], but we need further confirmation of these results. This study has uncovered evidence supporting the potential role of mediastinal fat in predicting the malignancy of pulmonary nodules. Furthermore, the results of NRI and IDI comparisons demonstrated a significant enhancement in predictive accuracy (*p* < 0.05), further emphasizing the substantial influence of mediastinal fat in predicting pulmonary nodule malignancy. These findings collectively suggested that adipose tissue contained valuable information reflecting the tumor microenvironment.

The deep learning radiomics model incorporates not only changes in nodule morphology but also radiomics features, reflecting the microscopic structures of the nodules [42]. We observed an enhanced diagnostic accuracy (C-index: 0.840) when comparing the model solely based on radiomics features with the model that integrated both deep learning and radiomics features. The quantitative features of the mediastinal fat region were integrated with those of the pulmonary nodule region, which further improved the diagnostic performance (C-index: 0.903). These findings are consistent with previous studies, demonstrating that the nomogram model combining radiomics and deep learning performed best in distinguishing between benign and malignant nodules.

Our study has several limitations. ROIs were delineated in a single layer (2D), potentially limiting their ability to fully represent the entire pulmonary nodule or the intrathoracic region. Hence, further research is warranted to explore the 3D analysis of the entire pulmonary nodule and intrathoracic fat. Furthermore, a prospective clinical trial is needed to establish the generalizability of our findings, as this investigation is retrospective in nature. Finally, because the underlying mechanisms of mediastinal fat in differentiating benign from malignant nodules remain unclear, more research is still required.

## 5. Conclusions

In conclusion, this study found that mediastinal adipose tissue was a valuable parameter for distinguishing between benign and malignant pulmonary nodules. Adding mediastinal fat tissue as a complement to intrathoracic information demonstrated promising performance in predicting the presence of malignant nodules. The deep learning-based radiomics nomogram model can serve as a non-invasive diagnostic tool for distinguishing between benign and malignant pulmonary nodules.

## Figures and Tables

**Table 1:** Patient characteristics in the training, validation, internal testing, and external testing sets.

Characteristic	Training Set	Validation Set	*p* Value	Internal Testing Set	External Testing Set 1	External Testing Set 2
**Number of patients**	594	199		199	182	220
**Age** **(yr, mean ± s.d)**	57.3 ± 9.1	57.2 ± 9.3	0.899	57.3 ± 9.5	57.9 ± 9.8	57.2 ± 9.8
**Sex**			0.840			
Female	360(60.6)	119(59.8)		125(62.8)	116(63.7)	132(60.0)
Male	234(39.4)	80(40.2)		74(37.2)	66(36.3)	88(40.0)
**Smoking**			0.752			
Yes	178(30.0)	62(31.2)		65(32.7)	36(19.8)	43(19.5)
No	416(70.0)	137(68.8)		134(67.3)	146(80.2)	177(80.5)
**Pathological type**			0.982			
**Malignant nodule**	481	161		162	154	155
Adenocarcinoma	449(93.5)	156(96.9)		155(95.7)	150(97.4)	144(92.9)
Squamous carcinoma	26(5.4)	4(2.5)		6(3.7)	2(1.3)	9(5.8)
Other Malignant histological types	6(1.2)	1(0.6)		1(0.6)	2(1.3)	2(1.3)
**Benign nodule**	113	38		37	28	65
Hyperplasia	38(33.6)	12(31.6)		9(24.3)	8(28.6)	10(15.4)
Hamartoma	21(18.6)	4(10.5)		10(27.0)	5(17.8)	11(16.9)
Other Benign histological types	54(47.8)	22(57.9)		18(48.7)	15(53.6)	44(67.7)

Note: The numbers in parentheses represent percentages. The *p* values were calculated using the *t* test for continuous variables and the χ^2^ test for categorical variables. The *t*-test was used to compare the means of continuous variables between the two groups to determine if their differences were statistically significant. The chi-square test was utilized for categorical variables to assess if there is a significant association between the groups, using a significance level of 0.05.

**Table 2:** Variables and coefficients of models.

Intercept and Variables	Model 1			Model 2			Model 3		
	β	OR (95%CI)	*p* Value	β	OR (95%CI)	*p* Value	β	OR (95%CI)	*p* Value
Intercept	−3.107	-	<0.001	−6.220	-	<0.001	−10.608	-	<0.001
Nodule.radiomic.score	5.977	394.278 (45.183–3440.535)	<0.001	4.880	131.644 (14.599–1187.028)	<0.001	4.263	71.006 (5.539–910.308)	0.001
Nodule.DL.score	-	-	-	5.305	201.255 (20.273–1997.909)	<0.001	5.182	178.108 (13.557–2339.988)	<0.001
Mediastinal.fat.score	-	-	-	-	-	-	6.411	608.687 (29.190–12,692.520)	<0.001

Note: β = regression coefficient, OR = odds ratio, CI = confidence interval. Model 1—radiomics model that includes only nodule region features; Model 2—deep learning radiomics model that includes only nodule region features; Model 3—model that combines nodule region and mediastinal fat features. β (Beta): Indicates the effect size of predictors in regression. OR shows the odds of an outcome occurring with a specific exposure versus without. CI defines the range within which the true value likely falls, often at a 95% confidence level. The *p* values determine the statistical significance; values below 0.05 suggest significant results against the null hypothesis.

**Table 3:** Performance of predicted models in all sets.

	Internal Testing Set			External Testing Set1			External Testing Set2		
	Model 1	Model 2	Model 3	Model 1	Model 2	Model 3	Model 1	Model 2	Model 3
**Overall**									
Brier	0.112	0.090	0.079	0.118	0.107	0.068	0.176	0.159	0.133
R^2^	0.299	0.443	0.560	0.154	0.258	0.591	0.201	0.308	0.484
**Discrimination**									
C-index	0.766	0.840	0.903	0.747	0.829	0.942	0.732	0.794	0.880
Specificity	0.676	0.784	0.919	0.679	0.857	0.929	0.708	0.708	0.908
Sensitivity	0.765	0.741	0.765	0.786	0.714	0.864	0.671	0.703	0.755
Discrimination slope	0.254	0.385	0.476	0.105	0.190	0.500	0.156	0.239	0.379
**Calibration**									
H-L test (*p*)	0.770	0.280	0.725	0.478	0.652	0.651	0.824	0.784	0.446
**Clinical usefulness**									
Accuracy	0.869	0.894	0.910	0.824	0.863	0.901	0.732	0.777	0.789

Note: Model 1—radiomics model that includes only nodule region features; Model 2—deep learning radiomics model that includes only nodule region features; Model 3—model that combines nodule region and mediastinal fat features. The Brier score assesses prediction accuracy; lower scores indicate better predictions. R^2^ reflects the proportion of variance in the dependent variable explained by independent variables; values closer to 1 suggest a better model fit. The C-index evaluates the predictive accuracy of risk models, particularly in survival analysis; higher values indicate better prediction capability.

**Table 4:** Model performance in the training and internal testing sets.

	Training Set			Internal Testing Set		
	NRI	95%CI	*p* Value	NRI	95%CI	*p* Value
Model 2 vs. Model 1	0.140	0.063–0.217	<0.001	0.177	0.029–0.325	0.019
Model 3 vs. Model 2	0.144	0.057–0.231	<0.001	0.243	0.104–0.383	<0.001
	**IDI**	**95%CI**	***p* Value**	**IDI**	**95%CI**	***p* Value**
Model 2 vs. Model 1	0.150	0.109–0.190	<0.001	0.132	0.058–0.205	<0.001
Model 3 vs. Model 2	0.130	0.089–0.171	<0.001	0.090	0.030–0.151	0.003

Note: CI = confidence interval, NRI = net reclassification improvement test, IDI = integrated discrimination improvement test. Model 1—radiomics model that includes only nodule region features; Model 2—deep learning radiomics model that includes only nodule region features; Model 3—model that combines nodule region and mediastinal fat features. NRI measures how well a new model reclassifies subjects into correct risk categories compared to a reference model. Positive NRI indicates better performance. IDI assesses improvement in model discrimination, specifically the ability to separate those with and without the event. Higher IDI values signify better model differentiation.

## Data Availability

All supporting data are contained within the article. The datasets used and/or analyzed in this study are available from the corresponding authors upon reasonable request.

## Supplementary Materials

The following supporting information can be downloaded at https://www.mdpi.com/article/10.3390/biomedicines12081865/s1, Supplementary File S1: Patient recruitment; Supplementary File S2: CT image acquisition; Supplementary File S3: Imaging data processing; Supplementary File S4: Radiomic feature extraction; Supplementary File S5: Deep learning feature extraction; Supplementary File S6: Evaluation of model performance; Supplementary File S7: Method of feature selection; Supplementary File S8: Stratified analysis of nomogram; Figure S1: The detailed process of extracting mediastinal fat mask from CT images; Figure S2: Network architecture of multi-scale channel attention mechanism; Figure S3: Network architecture of Swin transformer model; Figure S4: Pulmonary nodule texture features and mediastinal fat features selection using the least absolute shrinkage and selection operator (LASSO) logistic regression model; Figure S5: The clinical impact plot and the ROC component plot; Figure S6: Model 3 score for each subgroup; Table S1: The CT image acquisition parameters of the three centers; Table S2: Machine learning model selection for nomogram score building.
