# Supplementary material for: Deep Learning Radiomics Features of Mediastinal Fat and Pulmonary Nodules on Lung CT Images Distinguish Benignancy and Malignancy
Source: Biomedicines. 2024 Aug 15;12(8):1865. doi: 10.3390/biomedicines12081865 (PMC11352131; doi:10.3390/biomedicines12081865)
Supplement: Supplementary file 1 [file biomedicines-12-01865-s001.zip › biomedicines-3138866-supplementary.pdf]

Supplementary material online for

"Deep Learning Radiomics Features of Mediastinal fat and Pulmonary Nodules on Lung CT Images Distinguish Benignancy and Malignancy"

This supplementary material includes:

Supplementary File S1: Patient recruitment

Supplementary File S2: CT image acquisition

Supplementary File S3: Imaging data processing

Supplementary File S4: Radiomic feature extraction

Supplementary File S5: Deep learning feature extraction

Supplementary File S6: Evaluation of model performance

Supplementary File S7: Method of feature selection

Supplementary File S8: Stratified analysis of nomogram

References for Supplementary Material

Figure S1. The detailed process of extracting mediastinal fat mask from CT images

Figure S2. Network architecture of multi-scale channel attention mechanism

Figure S3. Network architecture of Swin transformer model

Figure S4. Pulmonary nodule texture features and mediastinal fat features selection using the least absolute shrinkage and selection operator (LASSO) logistic regression model

Figure S5. The clinical impact plot and the ROC component plot

Figure S6. Model 3 score for each subgroup

Table S1. The CT image acquisition parameters of the three centers

Table S2. Machine learning model selection for nomogram score building.

Supplementary File S1: Patient Recruitment

This retrospective study was a multi-center study involving 1394 patients from three hospitals. All patients were allocated into five sets: one training set, one validation set, one internal testing set, and two external testing sets. Detailed information about each set is provided below.

According to the 3:1:1 ratio, 992 patients from center 1 (Harbin Medical University Cancer Hospital) was randomly divided into a training set, an internal validation set, and a test set.

(1) Training set

The training set was recruited from December 2016 to June 2022 at Center 1 and consisted of 594 patients (male vs female: 234 vs 360, malignant vs benign: 481 vs 113, smoking yes vs no: 178 vs 416, mean age: 57.3 years).

(2) Validation set

The internal validation set was recruited from December 2016 to June 2022 at center 1 and consisted of 199 patients (male vs female: 80 vs 119, malignant vs benign: 161 vs 38, smoking yes vs no: 62 vs 137, mean age: 57.2 years).

(3) Internal testing set

The test set was recruited from December 2016 to June 2022 at center 1 and consisted of 199 patients (male vs female: 74 vs 125, malignant vs benign: 162 vs 37, smoking yes vs no: 65 vs 134, mean age: 57.3 years).

(4) External testing set 1

The external validation set 1 was recruited from January 2019 to February 2023 at center 2 (First Affiliated Hospital of Harbin Medical University) and consisted of 182 patients (male vs female: 66 vs 116, malignant vs benign: 154

vs 28, smoking yes vs no: 36 vs 146, mean age: 57.9 years).

(5) External testing set 2

The external validation set 2 recruited from January 2021 to February 2023 from center 3 (Second Affiliated Hospital of Harbin Medical University) and consisted of 220 patients (male vs female: 88 vs 132, malignant vs benign: 155 vs 65, smoking yes vs no: 43 vs 177, mean age: 57.2 years).

The inclusion and exclusion criteria were shown as follows.

The inclusion criteria were as follows: (a) subjects had to be older than 18 years; (b) all patients underwent lung nodule resection, and postoperative pathology was performed; (c) CT scan was carried out less than 2 weeks before surgery; (d) the CT measurement diameter of nodules ranges from 6 to 30 millimeters.

The exclusion criteria were the following: (a) preoperative treatment; (b) incomplete clinical data; (c) a lack of chest CT images or there are artifacts on the CT image that affect the evaluation; (d) had distant metastasis at diagnosis.

Supplementary File S2: CT image acquisition

Enrolled patients in the three centers underwent similar scan setups but with different systems and parameters. For contrast-enhanced CT scans, patients received CT examinations in the axial plane after the injection of a contrast agent via a pump injector into the antecubital vein. The CT scans, covering the entire pulmonary region, were acquired during a breath-hold with the patient supine. Detailed CT image acquisition parameters for the three centers were provided in Table S1. CT images from the transverse plane were retrieved from the Picture Archiving and Communication System (PACS) and were utilized in this study.

The diagnosis was performed with a standardized dynamic window adjustment procedure, on window adjustable PACS workstations. CT scans were independently reviewed by two chest radiologists with 30 (reader 1) and

10 (reader 2) years of experience, who were blinded to the pathologic diagnosis and medical history of the patients. The images were viewed in the pulmonary (level: -600 Hounsfield units [HU]; width: 1500 HU) and mediastinal windows (level: 40 HU; width: 300 HU). Consensus was reached through discussion.

The following subjective CT findings were noted: (a) the location of the pulmonary nodules (left upper lobe, left lower lobe, right upper lobe and right middle lobe, or right lower lobe); (b) the size of the lesion (the average of the major diameter and vertical short diameter measured on the section where the nodule appeared the largest in the pulmonary window); (c) the lesion margin (regular or irregular); (d) the presence of a lobulated shape (yes or no); (e) the presence of a spiculated sign (yes or no). Lobulated shape was defined if an abrupt bulging of the contour of the lesion was seen. Spiculated sign was defined if thicker strands were seen extending from the nodule margin into the pulmonary parenchyma without reaching the pleural surface [2].

Supplementary File S3: Imaging data processing

To ensure the precise capture of nodule areas, before training the Convolutional Neural Network (CNN), rectangular regions of interest (ROI-1) containing the nodules are cropped from CT images based on the coordinates of the pulmonary nodules. The research has obtained the original CT images and located the coordinates of the pulmonary nodules. The localization results are typically represented using the coordinates of the nodule center (nodule_center_x, nodule_center_y). Then, a 50×50-pixel nodule region image is cropped from the original CT image with the nodule center as the center point. To achieve this, the nodule center coordinates are used as a reference, and the image is offset 25 pixels to the left and upward and 25 pixels to the right

and downward, resulting in a 50×50-pixel square region. This ensures that the center of the nodule remains at the center of the cropped image. The cropped nodule images can be used as inputs to the ResNet architecture, where the rectangular regions of interest containing the pulmonary nodules are cropped from the CT images based on the coordinates of the pulmonary nodules and resized to 224×224 before training the CNN. This operation allows the regions of interest to adapt to the CNN structure.

As for the mediastinal fat area, Figure S1 illustrates the detailed process of extracting the mediastinal fat mask from the CT images. To obtain the mediastinal fat tissue region, a threshold of HU ranging from -200 to -40 is set in the CT images using Image J, resulting in the red region (a), representing all the fat tissues in the CT image (b). Next, based on (b), the mediastinal fat region of interest is manually delineated (c) with careful labeling by experienced medical experts or individuals under medical expert guidance to ensure accurate depiction of the mediastinal fat area and avoid the inclusion of other tissues or structures. Then, the manually delineated mediastinal fat region is intersected with the previously obtained total fat tissue region in the CT image to perform the intersection operation, resulting in the final mediastinal fat region. This intersection operation helps to eliminate errors or misdrawn parts, obtaining a more accurate representation of the mediastinal fat area, and further generating the corresponding mask (d). The mediastinal fat mask accurately indicates the location and scope of the mediastinal fat tissue in the

CT image, providing a foundation for the subsequent analysis and processing by deep learning models. It is essential to note that the entire process requires careful delineation and threshold adjustment to ensure accurate coverage of the mediastinal fat area and necessitates necessary checks to obtain the final mediastinal fat mask. To ensure the capture of the entire mediastinal fat region, before training the Swin Transformer, rectangular regions of interest (ROI-2) are cropped from the mediastinal fat mask image based on the body part and resized to 224×224. This operation allows the regions of interest to adapt to the CNN structure. Additionally, all rectangular regions of interest (ROIs) are standardized, so they follow the traditional normal distribution.

Supplementary File S4: Radiomic feature extraction

The method for extracting nodule radiomics features is implemented using the radiomics library in Python. The data consists of CT images with nodules centered on the largest diameter of the nodule, along with masks representing nodule regions (pixel value 255) and non-nodule regions (pixel value 0).

A filtering process was performed to implement image smoothing and image difference before CT radiomic feature extraction. Separable filtering was used to avoid the multi-dimensional convolution. The convolution was performed with a low-/high-pass "Coiflet 1" wavelet filter along the x-/y-direction, separately. Consider L and H to be low-pass and high-pass functions, respectively, X to be the original CT image, and the filtered results of X to be labelled as X_{LL}, X_{HH}. That is, two new images were obtained by filtering the

original image in two directions (x, y).

After filtering, a total of 289 nodule radiomics features were extracted from the ROI of the original image and its corresponding filtered results. These features can be categorized into three main groups: (1) First-order features: these features are directly computed from the Hounsfield intensity units, such as minimum, maximum, mean, and variance. (2) Shape features: shape features are used to quantify the 3D shape and size of the tumors, including volume and surface area. (3) Texture features: texture features are calculated based on the spatial relationships between voxels and include features derived from gray-level co-occurrence matrix (GLCM), gray-level run-length matrix (GLRLM), gray-level size zone matrix (GLSZM), gray-level dependence matrix, and gray-level difference matrix.

Nodule radiomics features of all patients were standardized by the z-score method, based on the parameters calculated from the training set.

Supplementary File S5: Deep learning feature extraction

Resnet18

ResNet18 introduces residual connections, where input features are added to the output features through skip connections across layers, making it easier for the model to learn residual components. In the context of pulmonary nodule feature extraction, residual connections help preserve crucial details and alleviate feature loss. Due to the existence of residual connections, ResNet18 allows information to flow freely within the network. This characteristic

promotes feature reuse, enabling earlier layers to communicate directly with subsequent layers through residual connections, thus passing low-level features to higher-level layers. In the context of pulmonary nodule feature extraction, this feature reuse facilitates the network in capturing the shape, texture, and edges of pulmonary nodules more effectively, improving feature consistency and stability.

Attention mechanism module

The study proposed a multi-scale relation-aware attention mechanism, and the introduction of the attention mechanism module aims to improve the accuracy of pulmonary nodule prediction. The input feature map is divided into two scales along the channel dimension. For each segmented part, it has two shared channels, and the i*th* feature map is represented as $X_i \in R^{C_i \times H \times W}$. Individual features are fused before passing to the relation-aware module, which consists of the channel attention module for semantic segmentation.

The second part of our network performs the following steps:

(1) Constructs a channel attention matrix to model the relationships between any two channels of the features.

(2) Conducts matrix multiplication between the attention matrix and the original features.

(3) Performs element-wise summation between the obtained matrix and the original features.

(4) Applies a softmax layer to obtain the channel attention mapping $X \in R^{C \times C}$.

The network architecture of the multi-scale channel attention mechanism is shown in Figure S2.

Resnet18 model parameter

In this study, the central dataset was randomly divided into a training set and a validation set in a ratio of 3:1. A CNN model was used as a deep learning feature extractor. During the development phase, ROIs were input into the network model, and the model parameters were updated through backpropagation. To reduce overfitting, image augmentation was performed using random horizontal and vertical flips before inputting the data. The BCEWithLogitsLoss function was used as the model's loss function. The training parameters were as follows: Adam optimizer with a batch size of 32, initial learning rate of 0.0001, maximum number of iterations set to 200, and the learning rate was decayed by 50% every 200 steps.

Swin Transformer

Swin Transformer is a Transformer-based image classification model that introduces a hierarchical window-based self-attention mechanism to handle large-scale images more efficiently. The input pulmonary nodule images are first divided into small image blocks. Unlike traditional Vision Transformers, Swin Transformer uses fixed-size small image blocks for segmentation instead of overlapping windows. These image blocks undergo path extraction through a series of small Transformer blocks. These small Transformer blocks are divided into different stages, with each stage containing multiple blocks. The

goal of each stage is to learn local contextual features for each position in the image blocks and capture the dependencies between positions. Each small Transformer block utilizes a window-based self-attention mechanism, where image blocks are partitioned into windows, and self-attention is applied within each window. This reduces computational complexity and enhances model scalability while combining local and global contextual information of image features. The Swin Transformer employs a pooling stage to aggregate the outputs of each small Transformer block. This stage aggregates and enhances image block-level features through a series of transposed convolutions and downsampling operations divided into multiple stages. The Swin Transformer also introduces conventional Transformer encoder modules, which model global context across the entire image and compute image representations. Finally, the global image representation undergoes a fully connected layer for pulmonary nodule benign/malignant prediction. In each stage, a Patch Merging layer is applied for downsampling. Suppose the input to the Patch Merging layer is a 4×4-sized single-channel feature map. The Patch Merging layer divides each 2×2 adjacent pixels into a patch, and the pixels in each patch are then concatenated together to obtain 4 feature maps. These four feature maps are further concatenated in the depth direction and passed through a LayerNorm layer. Finally, a fully connected layer performs a linear transformation in the depth direction, reducing the depth of the feature map from C to C/2. From Figure S3, it can be observed that after the Patch Merging

layer, the height and width of the feature map are halved, and the depth is doubled. The model flow of Swin Transformer is illustrated in Figure S3.

Swin Transformer model parameter

After loading pre-trained weights from ImageNet, we performed a two-step fine-tuning training process for the Swin Transformer. Specifically, except for the last layer, all layers were initialized using pre-trained weights. To conduct fine-tuning, the training dataset consisted of thousands of 224×224 images extracted from CT images of the primary population, ensuring that the mediastinal fat ROI covered at least one-third of each image. Data augmentation strategies such as rotation, mirroring, and adding Gaussian noise were employed. We used mean squared error as the loss function and optimized the Swin Transformer using the Adamw optimizer. Each batch contained 32 images. In the first step of fine-tuning, we kept the convolutional layers frozen and trained only the last layer with a relatively small initial learning rate (0.00001). In the second step, we trained all layers and further reduced the initial learning rate to one-tenth of that used in the first step. L1-L2 regularization was applied to the last layer to control overfitting and improve the representation capacity of the convolutional layers.

Supplementary File S6: Evaluation of model performance

First, we compared the overall performance of each model used in this study using Brier score and Nergekerke's R^2. We assessed the models' discriminative ability using C-index and discriminant slope. A C-index greater

than 0.8 indicates good model performance . The discriminant slope represents the separation between benign and malignant patients. Additionally, methods like Integrated Discrimination Improvement (IDI) and Net Reclassification Improvement (NRI) provided a direct way to quantify the improvement in model performance due to the biomarker. Calibration curves were employed to calibrate the models, which illustrate how well the predicted probabilities match the observed malignancy of pulmonary nodules in the study. Simultaneously, the Hosmer-Lemeshow goodness-of-fit test was applied to evaluate whether the model's expected probabilities fit the actual probabilities. To determine if the predictive models are applicable in clinical practice, we further quantified the clinical utility of the models through Decision Curve Analysis (DCA), clinical impact analysis, and Receiver Operating Characteristic (ROC) curve component analysis. These evaluations provide insights into the models' performance, clinical applicability, and their ability to distinguish between benign and malignant pulmonary nodules, helping to assess their potential value in real-world clinical scenarios.

Supplementary File S7: Method of feature selection

In this study, feature selection was performed for both the radiomic features of pulmonary nodules and the mediastinal fat features. The Least Absolute Shrinkage and Selection Operator (LASSO) regression model was used for feature selection separately on these two types of data. LASSO is a common method for high-dimensional predictive factor regression. The study

applied L1 penalty to shrink some regression coefficients exactly to zero. We plotted the AUC and log(λ), where λ represents the tuning parameter of the LASSO logistic regression model. To avoid overfitting, we performed 10-fold cross-validation and selected the simplest model while maintaining the predictive performance as high as possible. A red vertical line was plotted at λ, corresponding to the optimal value. In the feature selection process for the radiomic features of pulmonary nodules, the optimal tuning parameter resulted in 10 non-zero coefficients in the final logistic regression model (λ = 0.06375318, log(λ) = -2.752736). The study extracted 289 features from the radiomic characteristics of pulmonary nodules.

Used the penultimate fully connected (FC) layer of ResNet18 as the feature output for each CT image. Subsequently, a multivariate logistic regression was employed to combine the selected features and construct network features. Ten features were extracted from the CT images of pulmonary nodules and named as $x_i, i \in [1,10]$. Additionally, for the mediastinal fat features, the optimal tuning parameter resulted in 9 non-zero coefficients in the final logistic regression model (λ = 0.02787933, log(λ) = -3.57987). As shown in Figure S4. 768 features were extracted from the mediastinal fat mask using the Swin Transformer model and named as $y_i, i \in [1,768]$. After feature selection, a total of 10 radiomic features, 10 deep learning network features, and 9 mediastinal fat features were obtained for pulmonary nodules. Subsequently, the score was established by comparing the three machine methods, as shown in Table S2. Finally,

multivariate logistic regression was used to combine the selected features and construct Nodule.radiomic.score, Nodule.DL.score, and Mediastinal.fat.score. The model was developed using logistic regression techniques. The score was calculated using the following formula:

$$S = \text{sigmoid}(\mathrm{w}_1\mathrm{x}_1 + \mathrm{w}_2\mathrm{x}_2 + \cdots + \mathrm{w}_i\mathrm{x}_i + \mathrm{b})$$

Where x_i is the selected features and w_i is the corresponding weight.

Supplementary File S8: Stratified analysis of nomogram

The generalizability of a nomogram is always a matter of great concern in terms of its practical applicability. Age was believed to influence the performance. In order to test the generalization ability of our nomogram, we performed stratification analysis on the subgroups of age, sex, version of CT and CT image thickness. We used the ROC curve and AUC to evaluate the performance of our nomogram on these subpopulations. The results showed that our nomogram was not influenced by these factors (all p>0.05) (see Figure S6), suggesting strong generalizability of the nomogram.

1. Stratified analysis on age： Patients are divided into two subgroups: age < 60 and age ≥ 60 with AUC of 0.943 and 0.909 (Delong test, p value: 0.124 and 0.708 compared with the result on the overall set).

2. Stratified analysis on sex： Patients are divided into two subgroups: male and female with AUC of 0.945 and 0.914 (Delong test, p value: 0.090 and 0.889 compared with the result on the overall set).

3. Stratified analysis on version of CT： Patients are divided into two subgroups: scanned using GE CT system and using SIEMENS CT system with AUC of 0.925 and 0.943 (Delong test, p value: 0.652 and 0.175 compared with the result on the overall set).

4. Stratified analysis on CT image thickness： Patients are divided into two subgroups: image thickness ≥5 mm and image thickness < 5 mm with AUC

of 0.937 and 0.922 (Delong test p value: 0.253 and 0.803 compared with the result on the overall set).

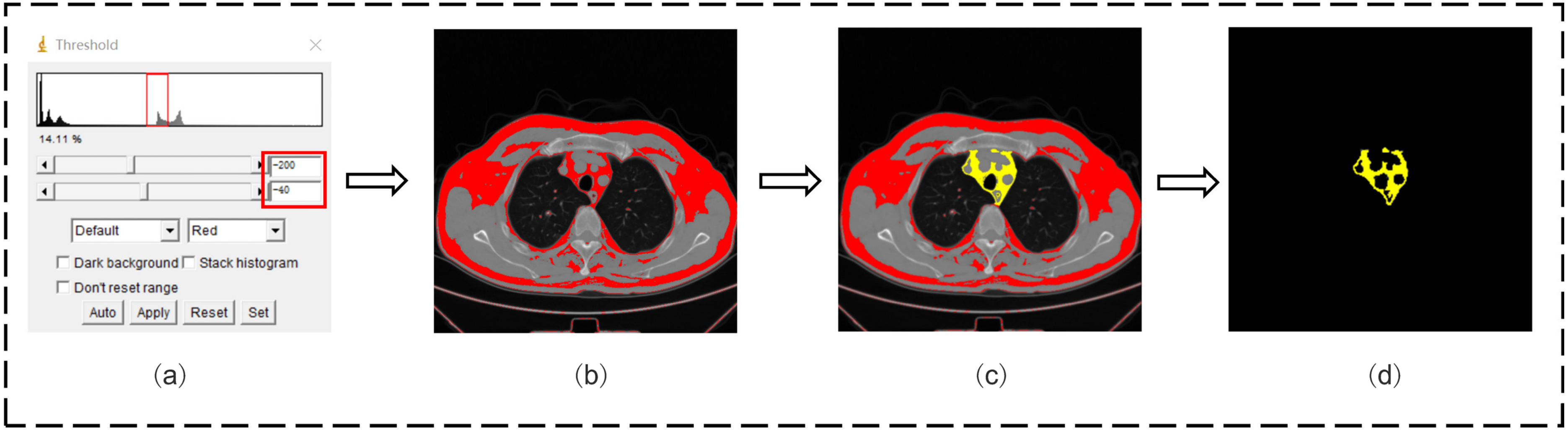

Figure S1. The detailed process of extracting mediastinal fat mask from CT images. (a) illustrates the threshold adjustment using Image-J, (b) displays all the fat tissues present in the image. (c) shows the intersection, resulting in the mediastinal fat region. Finally, (d) represents the generated Mask for the mediastinal fat region.

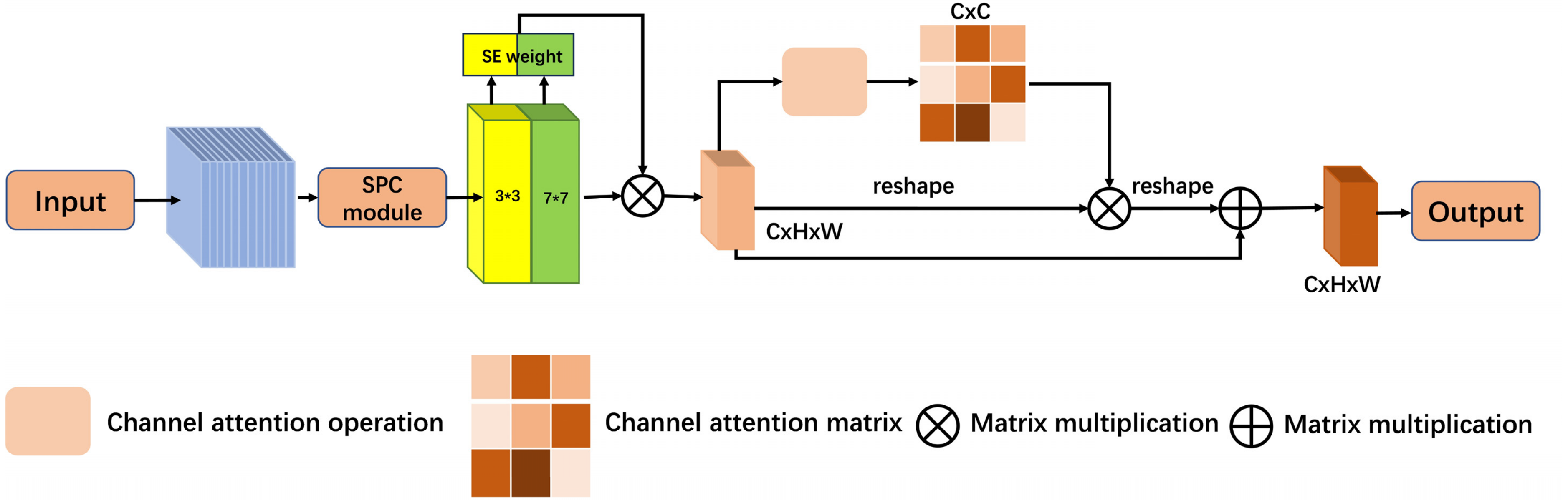

Figure S2. Network architecture of multi-scale channel attention mechanism. SPC: Squeeze and Concat module; SE weight: using the SE module to extract the multi-scale channel weight.

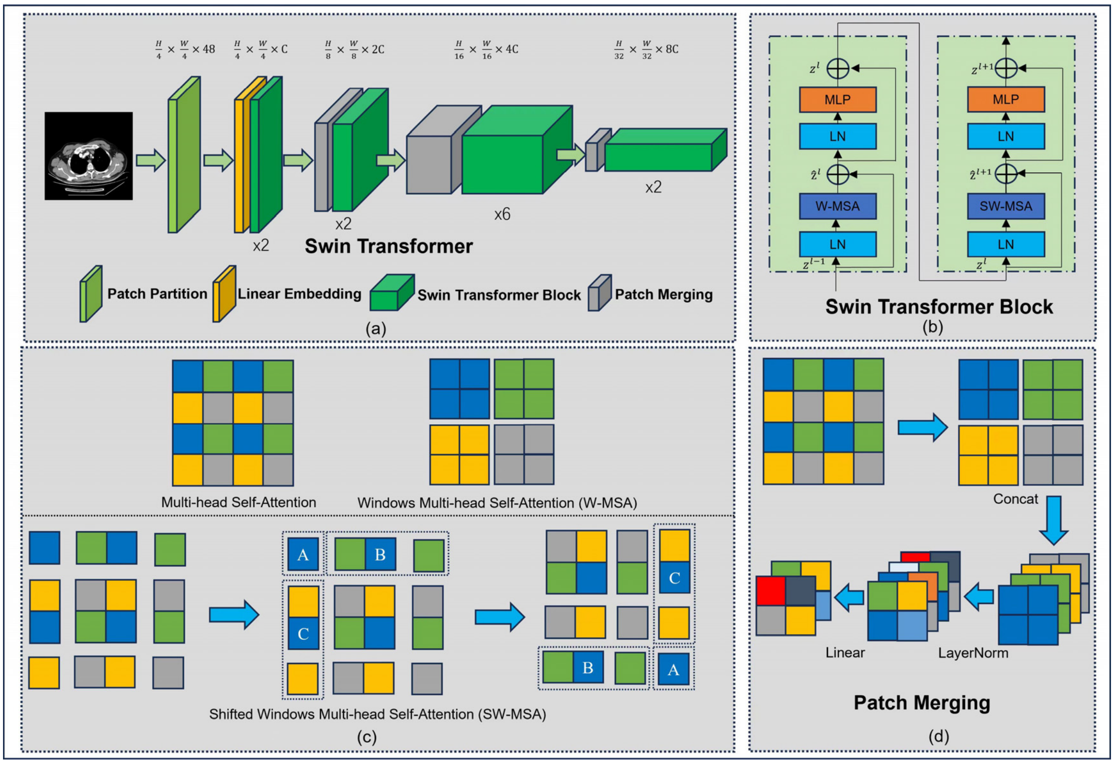

Figure S3. Network architecture of Swin transformer model. (a) the main architecture of the model, (b) the swin transformer bolck module, (c) several attention mechanisms and (d) patch merging module.

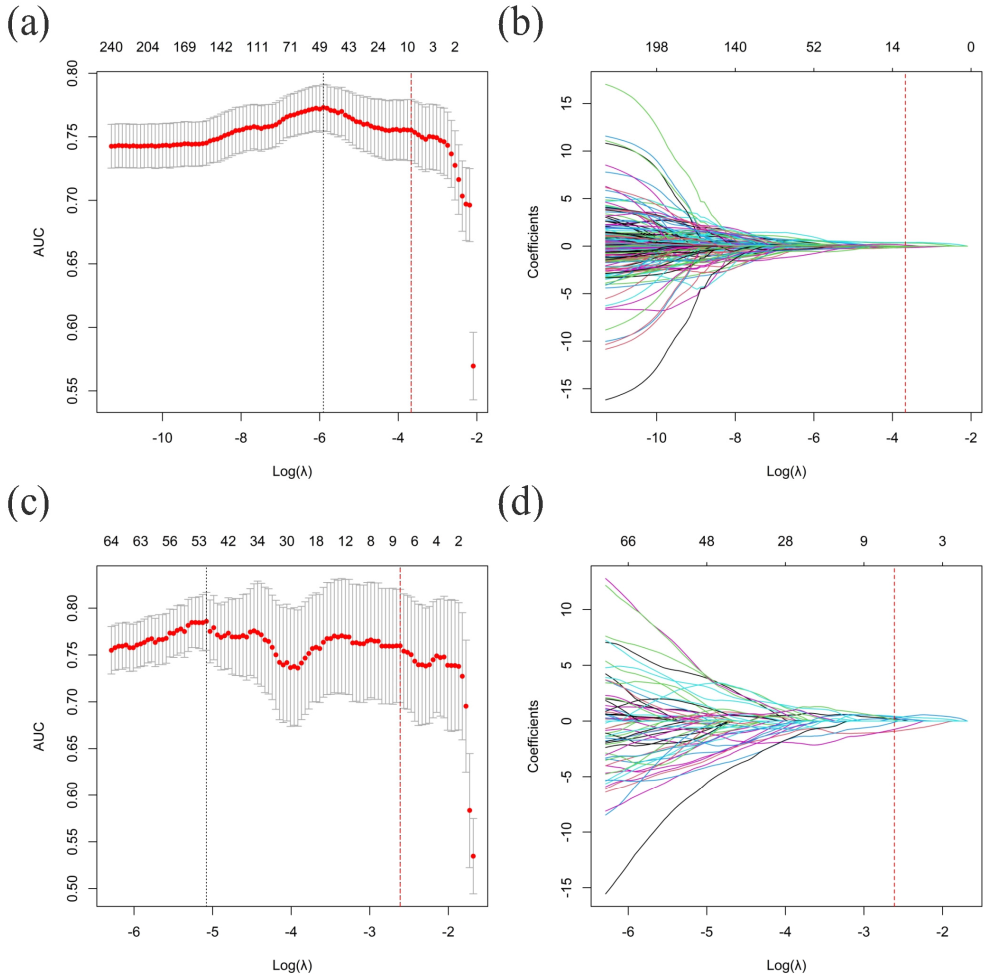

Figure S4. Pulmonary nodule texture features and mediastinal fat features selection using the least absolute shrinkage and selection operator (LASSO) logistic regression model. (a) Pulmonary nodule texture features tuning parameter (λ) selection in the LASSO model used via 10-fold cross-validation. The AUC curve was plotted versus log (λ). (b) LASSO coefficient profiles of the 289 pulmonary nodule texture features. A coefficient profile plot was produced against the log (λ) sequence. A vertical line was drawn at the value selected using 10-fold cross-validation, where optimal λ resulted in ten nonzero coefficients.(c) Mediastinal fat features tuning parameter (λ) selection in the LASSO model used via 10-fold cross-validation. The AUC curve was plotted

versus log (λ). (d) LASSO coefficient profiles of the 768 mediastinal fat features. A coefficient profile plot was produced against the log (λ) sequence. A vertical line was drawn at the value selected using 10-fold cross-validation, where optimal λ resulted in nine nonzero coefficients.

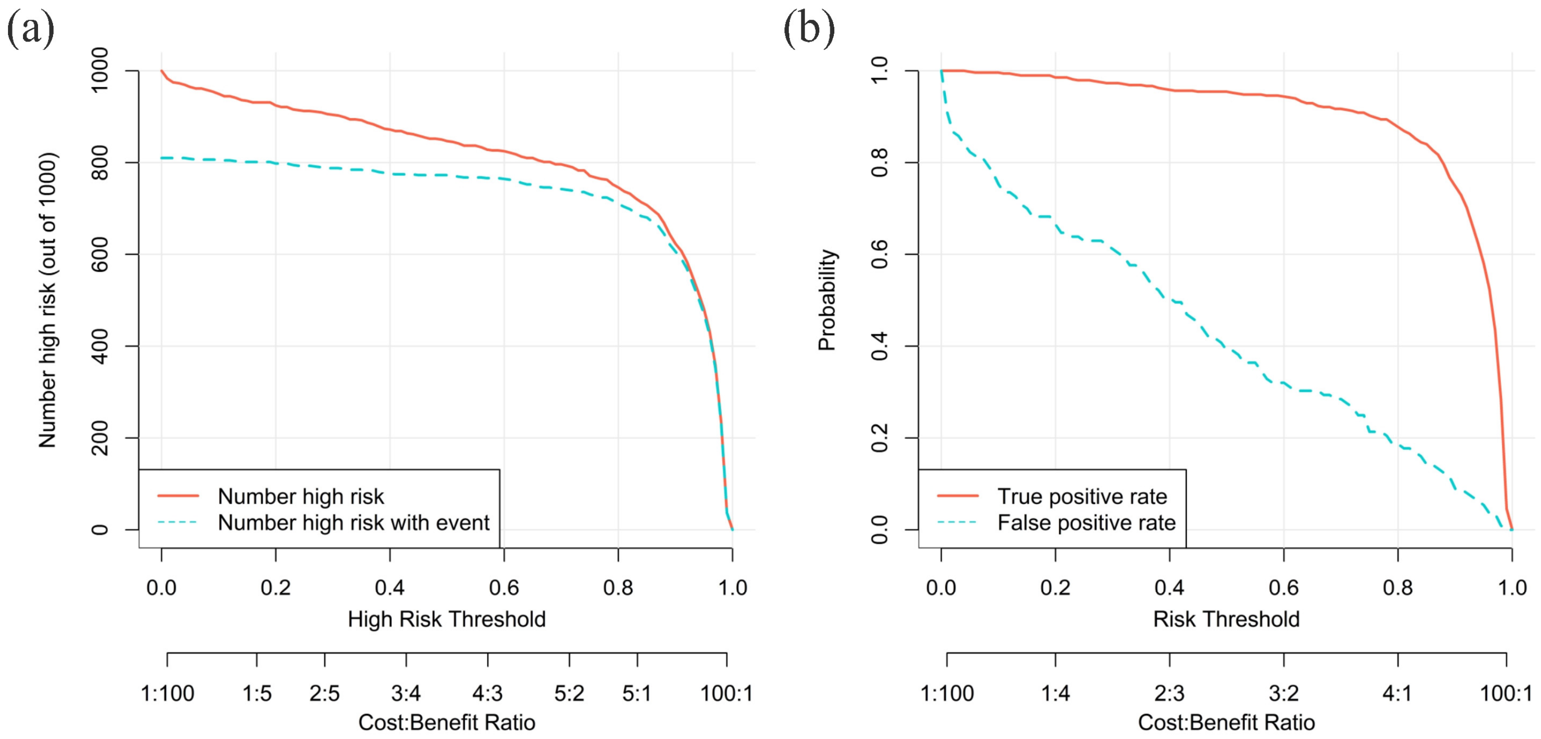

Figure S5. The clinical impact plot and the ROC component plot. (a) is the clinical impact curve of 1000 randomized patients in Model 3. The solid red line indicates the number of nodules considered malignant at the relevant risk threshold, and the dashed blue line indicates the actual malignant nodules. (b) is the true positive rate and false positive rate of model 3. The red solid line represents the true positive rate in each risk threshold, and the blue dashed line represents the false positive rate. Model 3, model that combines

nodule region and mediastinal fat features.

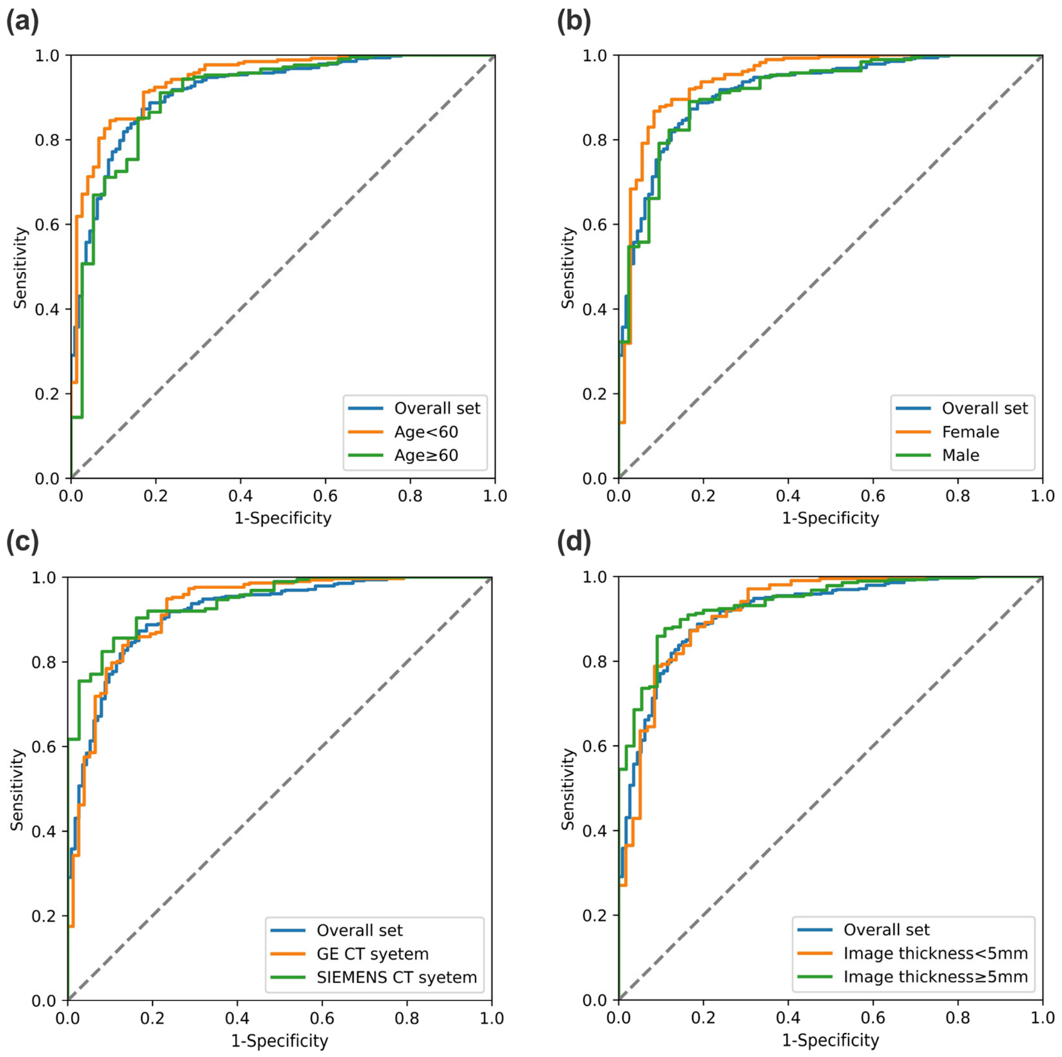

Figure S6. Model 3 score for each subgroup stratified by (a) age, (b) sex, (c) version of CT and (d) CT image thickness. Model 3, model that combines nodule region and mediastinal fat features.

Table S1. The CT image acquisition parameters of the three centers.

Parameters		Center 1	Center 2	Center 3
		Harbin Medical University Cancer Hospital	The Second Affiliated Hospital, Harbin Medical University	The First Affiliated Hospital, Harbin Medical University
**CT system information**	CT system	256-slice spiral CT (SOMATOM Definition Flash, Siemens Healthineers, Germany) or a 64-slice spiral CT (Brilliance 64, PHILIPS, Netherlands) or a 16-slice spiral CT (BrightSpeed, GE Medical Systems, USA)	256-slice spiral CT (Brilliance iCT, ROYAL PHILIPS, Netherlands) or a 64-slice spiral CT (Discovery CT750 HD scanner, GE Medical Systems, USA) or a 16-slice spiral CT (SOMATON Sensation 10, Siemens Healthineers, Germany)	256-slice spiral CT (Brilliance iCT, ROYAL PHILIPS, Netherlands) or a 64-slice spiral CT (Brilliance 16, PHILIPS, Netherlands)
**CT scan parameters**	Tube voltage	120 kVp	120 kVp	120 kVp
	Tube current	200-400 mA	150-300 mA	200-400 mA
	Rotation time	0.5 s	0.5 s	0.5 s
	Detector collimation	128×0.625 mm or 64×0.625 mm or 32×0.6 mm	128×0.625 mm or 64×0.625 mm or 32×0.6 mm	128×0.625 mm
	Arterial phase CT	35 s after injection	25-35 s after injection	28 s after injection
	Venous phase CT	60 s after injection	55-65 s after injection	60 s after injection
	With unenhanced CT	Yes	Yes	Yes
**CT image information**	Image matrix	512×512	512×512	512×512
	Field of view	400×400 mm or 500×500 mm	400×400 mm or 500×500 mm	400×400 mm
	Reconstruction section thickness	1.25 mm for enhanced CT<br>5 mm for both enhanced and unenhanced CT	1.25 mm for enhanced CT<br>5 mm for both enhanced and unenhanced CT<br>0.625 mm for enhanced CT	5 mm for both enhanced and unenhanced CT

Note：CT = Computed tomography.

Table S2. Machine learning model selection for nomogram score building.

Classifiers	AUC	ACC	SEN	SPE
RF	0.842<br>[0.824, 0.860]	0.860<br>[0.842, 0.878]	0.804<br>[0.786, 0.823]	0.765<br>[0.738, 0.774]
LG	0.870<br>[0.851, 0.889]	0.882<br>[0.864, 0.901]	0.865<br>[0.847, 0.884]	0.844<br>[0.826, 0.863]
DT	0.756<br>[0.738, 0.775]	0.789<br>[0.770, 0.808]	0.829<br>[0.811, 0.848]	0.708<br>[0.689, 0.727]

Note：95% confidence intervals are included in brackets.

AUC = area under the receiver operating characteristic curve, ACC = accuracy, SEN = sensitivity, SPE = specificity, RF = random forests, LG = logistic regression, DT = decision tree.
